# Supplementary material for: Forecasting the monthly incidence rate of brucellosis in west of Iran using time series and data mining from 2010 to 2019
Source: PLoS One. 2020 May 12;15(5):e0232910. doi: 10.1371/journal.pone.0232910 (PMC7217463; doi:10.1371/journal.pone.0232910)
Supplement: Attachment — Submitted filename: Responce to reviewers.doc [file pone.0232910.s002.doc]

Dear Editor,

Thank you for your constructive and valuable comments. Below we have provided a point-by-point response to all the comments.

**Comments to the Author**

The authors compared the performance of four machine learning methods in forecasting human brucellosis. Generally, the subject is interesting and the manuscript is well written. In my concern, there are some minor issues with the manuscript as follows:

Are there other disease that could look like the case definition? How sensitive and specific to Brucellosis is that definition.

No, there aren't. The clinical – epidemiological symptoms of brucellosis were considered disease cases including : fever, myalgia and para-clinical symptoms are very non-specific and it may be like to several disease, but the definition of definitive case in our study was the positive results of two routine lab tests for brucellosis) including, Wright’s (diagnostic test for brucellosis; values greater than 1.8 indicate presence of infection) and 2ME (Mercaptoethanol Brucella agglutination test) (brucellosis confirmatory test, which if greater than or equal to 1.4 is indicative of the presence of infection. Please refer to Page 13^th^ lines: 132-35

Do the patients have to have all of the signs at one time?

Not necessarily, because this is the prospective cohort studies, that may be people affect the brucellosis in various time points.

Do the authors mean random forest by “random accumulation”? Please correct them as random accumulation is not the usual term.

Thanks revised.

According to golden rules of reporting the numbers (BMJ publication) the numbers under 10, must be presented in letters!

Thanks revised.

The majority of the references (References: 5, 6, 20 and 22) are not up to date.

Thanks, revised.

All formulas must be numbered. Thanks, revised. Pages 5-6

Thanks revised.

Reviewer #2: Overall, I think this manuscript is well written. Just a few suggestions for the result section.
1. Table 1 and Table 2, add p values to compare the incidence between each characteristics.

Thanks, revised. Please see table 1 and 2

2. Add a time series correlation matrix or plot to show the correlation between the characteristics in Table 4 and the time series of brucellosis.

The table added. Page 22

3 Explain in detail how you optimize the parameters in your four machine learning models. For example, how did you choose the number of hidden layers for neural network. Please also list those parameters for the other models, and explain how did you determine these parameters.

Edited. Page 8^th^ lines 198-210 as follow as:

To implement the models, variables in Table 1 as well as climatic variables of wind speed (m/s) and temperature (Centigrade) were used as predictors and the numbers of brucellosis cases observed was used as the output….

4 In the discussion section, please include more discussion how to apply these models in real infectious disease surveillance.

Thanks, revised, as follow as: Page 13th Lines: 372-75
